# Supplementary material for: Linezolid Serum Concentration Variability Among Critically Ill Patients Based on Renal Function and Continuous Renal Replacement Therapy Administration
Source: Antibiotics (Basel). 2025 Nov 21;14(12):1188. doi: 10.3390/antibiotics14121188 (PMC12729784; doi:10.3390/antibiotics14121188)

Supplementary Table S1: CRRT settings and parameters

Patient	CRRT type	Machine	Blood flow (ml/min)	Citrate flow (ml/h)	Effluent (ml/h)	Reinfusate	Reinfusate flow (ml/h)	Q_{uf} (ml/h)	Residual diuresis (ml/die)
**Pt1**	CVVH	Prismamax	150	1500	-80	MultibicK2	1500	3080	75
**Pt2**	CVVH	Prismamax	130	1300	0	Phoxilium	1200	2500	2000
**Pt3**	CVVH	Prismamax	150	1500	0	Phoxilium	1500	3000	1900
**Pt4**	CVVH	Prismamax	130	1300	-150	Multibic K2	1300	2750	Anuria
**Pt5**	CVVH	Prismamax	150	1500	-100	Phoxilium	1500	3100	2700
**Pt6**	CVVH	Prismamax	130	1300	-200	Multibic K2	1200	2700	32
**Pt7**	CVVH	Prismamax	150	1500	0	Multibic K2	1500	3000	1800
**Pt8**	CVVH	Prismamax	150	1500	-20	Phoxilium	1600	3120	135
**Pt9**	CVVH	Prismamax	150	1500	-30	Phoxilium	1600	3130	Anuria
**Pt10**	CVVH	Prismamax	130	1300	-220	Multibic K2	1200	2720	Anuria
**Pt11**	CVVH	Prismamax	130	1300	0	Multibic K2	1200	2500	Anuria
**Pt12**	CVVH	Prismamax	150	1500	-100	Phoxilium	1500	3100	Anuria
**Pt13**	CVVH	Prismamax	130	1600	-40	Phoxilium	1300	2640	Anuria
**Pt14**	CVVH	Prismamax	170	1700	-100	Multibic K2	1800	3600	68
**Pt15**	CVVH	Prismamax	130	1300	0	Phoxilium	1200	2500	347
**Pt16**	CVVH	Prismamax	110	1300	-30	Phoxilium	1300	2630	400
**Pt17**	CVVH	Prismamax	150	1200	-110	Multibic K2	1200	2510	Anuria
**Pt18**	CVVH	Prismamax	150	1500	-125	Multibic K2	1500	3125	172
**Mean**	/	/	142	1378	-72,5	/	1394	2939	966

Abbreviations: CRRT = Continuous Renal Replacement Therapy, CVVH = Continuous Veno-Venous Hemofiltration

Supplementary Table S2: Comparison between renal function and CRRT groups.

	**≤30 ml/min (N=11)**	**CRRT (N=18)**	**31-70 ml/min (N=11)**	**71-120 ml/min (N=8)**	**>120 ml/min (N=6)**	**Overall (N=54)**
**C_{min-ss} (mg/L)**						
Mean (SD)	14.5 (12.2)	5.42 (3.91)	3.52 (2.55)	3.05 (4.25)	1.07 (0.763)	6.06 (7.57)
Median [Q1, Q3]	10.0 [7.05, 19.3]	4.60 [2.98, 7.35]	3.10 [1.85, 5.35]	1.65 [0.300, 3.55]	0.850 [0.475, 1.75]	3.90 [1.43, 7.20]
[Min - Max]	[1.30 - 40.0]	[0.80 - 17.0]	[0.30 - 7.20]	[0.30 - 12.8]	[0.30 - 2.00]	[0.30 - 40.0]
**eGFR (ml/min)**						
Mean (SD)	17.8 (7.82)	NA	56.5 (10.2)	88.0 (14.8)	158 (54.4)	68.6 (53.1)
Median [Q1, Q3]	18.0 [10.5, 25.0]	NA	56.0 [51.5, 63.0]	82.0 [76.8, 100]	139 [128, 153]	58.0 [26.5, 88.0]
[Min - Max]	[7.00 - 28.0]	NA	[38.0 - 70.0]	[73.0 - 114]	[122 - 266]	[7.00 - 266]
**BMI (Kg/m^2)**						
Mean (SD)	25.7 (4.19)	29.4 (8.35)	26.1 (3.36)	26.3 (2.72)	26.3 (2.69)	27.2 (5.7)
Median [Q1, Q3]	24.9 [24.3, 27.4]	27.8 [24.4, 30.8]	27.3 [23.9, 28.1]	26.2 [24.5, 27.9]	26.0 [24.4, 28.6]	26.3 [24.2, 28.6]
**Linezolid (mg/die)**						
1200	11 (100%)	18 (100%)	11 (100%)	8 (100%)	6 (100%)	54 (100%)
**Overdosing**						
N (%)	8 (72.7%)	4 (22.2%)	2 (18.2%)	1 (12.5%)	0 (0%)	15 (27.8%)
**In Range**						
N (%)	1 (9.1%)	11 (61.1%)	5 (45.5%)	3 (37.5%)	2 (33.3%)	22 (40.7%)
**Underdosing**						
N (%)	2 (18.2%)	3 (16.7%)	4 (36.4%)	4 (50.0%)	4 (66.7%)	17 (31.5%)
**Extended inf. (3h)**						
N (%)	5 (45.5%)	12 (67%)	8 (73%)	6 (75%)	4 (66.6%)	35 (65%)
**Continuous inf.**						
N (%)	6 (54.5%)	6 (33%)	3 (27%)	2 (25%)	2 (33.4%)	19 (35%)

Abbreviations: BMI = Body Mass Index, CRRT = Continuous Renal Replacement Therapy, eGFR = estimated Glomerular Filtration Rate

Supplementary Figure S1: Comparison between extended infusion (3 hours) and continuous infusion; A) Overall population; B) CRRT and non-CRRT patients.

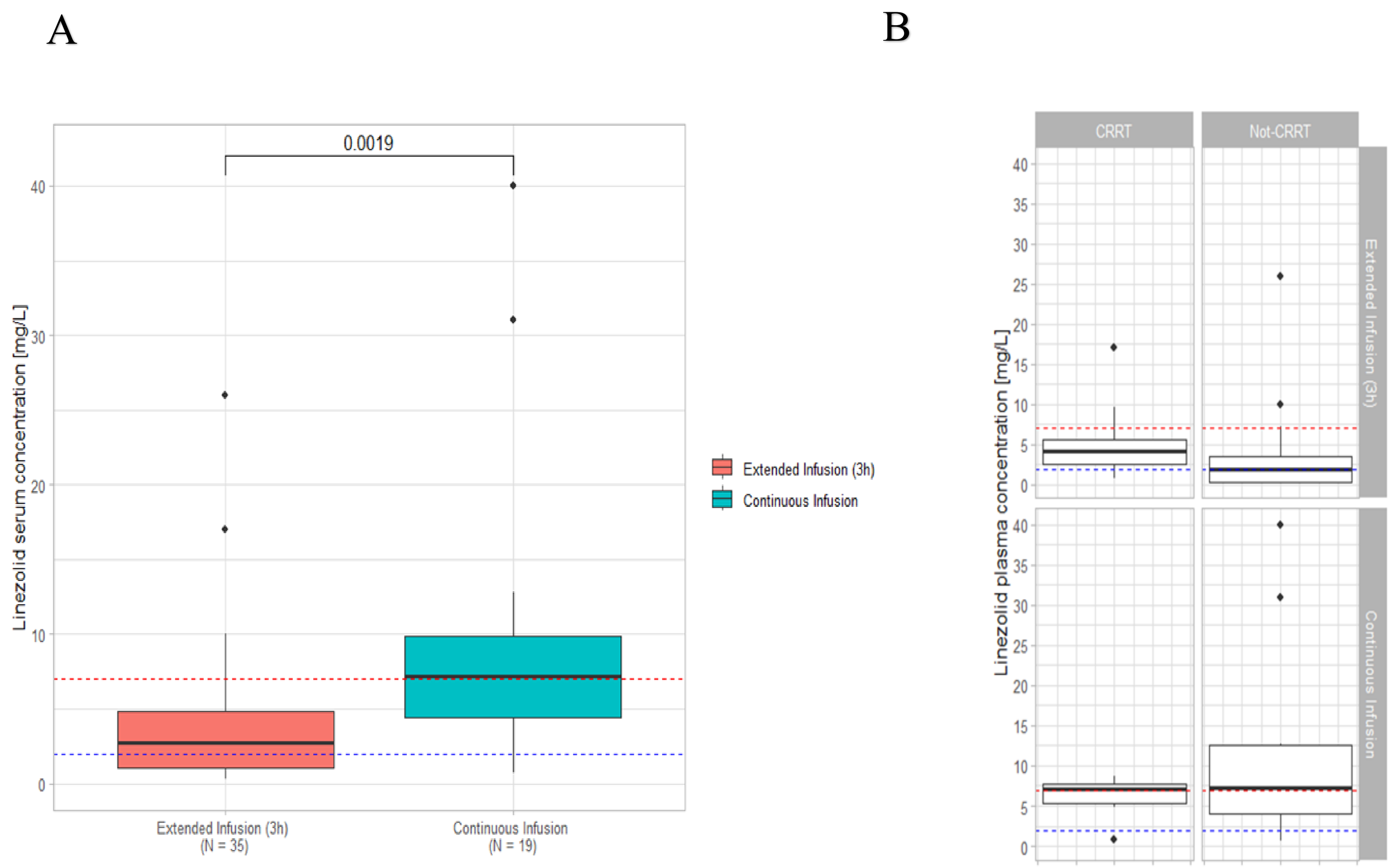

Supplement: Supplementary file 1 [file antibiotics-14-01188-s001.zip › antibiotics-3906809-supplementary.pdf]
